# Perinatal analyses of Zika- and dengue virus-specific neutralizing antibodies: A microcephaly case-control study in an area of high dengue endemicity in Brazil

**DOI:** 10.1371/journal.pntd.0007246

**Published:** 2019-03-11

**Authors:** Priscila M. S. Castanha, Wayner V. Souza, Cynthia Braga, Thalia Velho Barreto de Araújo, Ricardo A. A. Ximenes, Maria de Fátima P. M. Albuquerque, Ulisses R. Montarroyos, Demócrito B. Miranda-Filho, Marli T. Cordeiro, Rafael Dhalia, Ernesto T. A. Marques, Laura C. Rodrigues, Celina M. T. Martelli, Microcephaly Epidemic Research Group

**Affiliations:** 1 Aggeu Magalhães Institute, Oswaldo Cruz Foundation (FIOCRUZ), Recife, Pernambuco, Brazil; 2 School of Medical Science, University of Pernambuco, Recife, Pernambuco, Brazil; 3 Federal University of Pernambuco, Recife, Pernambuco, Brazil; 4 Department of Infectious Diseases and Microbiology, University of Pittsburgh, Pittsburgh, Pennsylvania, United States of America; 5 Department of Infectious Disease Epidemiology, London School of Hygiene & Tropical Medicine, London, United Kingdom; Faculdade de Medicina, Universidade Federal da Bahia, BRAZIL

## Abstract

Laboratory confirmation of Zika virus (ZIKV) infection during pregnancy is challenging due to cross-reactivity with dengue virus (DENV) and limited knowledge about the kinetics of anti-Zika antibody responses during pregnancy. We described ZIKV and DENV serological markers and the maternal-fetal transfer of antibodies among mothers and neonates after the ZIKV microcephaly outbreak in Northeast Brazil (2016). We included 89 microcephaly cases and 173 neonate controls at time of birth and their mothers. Microcephaly cases were defined as newborns with a particular head circumference (2 SD below the mean). Two controls without microcephaly were matched by the expected date of delivery and area of residence. We tested maternal serum for recent (ZIKV genome, IgM and IgG3 anti-NS1) and previous (ZIKV and DENV neutralizing antibodies [NAbs]) markers of infection. Multiple markers of recent or previous ZIKV and DENV infection in mothers were analyzed using principal component analysis (PCA). At delivery, 5.6% of microcephaly case mothers and 1.7% of control mothers were positive for ZIKV IgM. Positivity for ZIKV IgG3 anti-NS1 was 8.0% for case mothers and 3.5% for control mothers. ZIKV NAbs was slightly higher among mothers of cases (69.6%) than that of mothers of controls (57.2%; p = 0.054). DENV exposure was detected in 85.8% of all mothers. PCA discriminated two distinct components related to recent or previous ZIKV infection and DENV exposure. ZIKV NAbs were higher in newborns than in their corresponding mothers (p<0.001). We detected a high frequency of ZIKV exposure among mothers after the first wave of the ZIKV outbreak in Northeast Brazil. However, we found low sensitivity of the serological markers to recent infection (IgM and IgG3 anti-NS1) in perinatal samples of mothers of microcephaly cases. Since the neutralization test cannot precisely determine the time of infection, testing for ZIKV immune status should be performed as early as possible and throughout pregnancy to monitor acute Zika infection in endemic areas.

## Author summary

The epicenter of the ZIKV epidemic was located in Northeast Brazil (2015/16) and was followed by a space and time cluster of congenital microcephaly cases. This region is also a dengue hyperendemic setting. Laboratory confirmation of ZIKV infection during pregnancy is challenging due to cross-reactivity with other flaviviruses, especially dengue. The neutralization test, which is the gold standard to discriminate between these viruses, is time-consuming, performed in few laboratories and does not define the time when the infection occurred. This study described the serological markers of ZIKV and DENV among participants (mothers and neonates) of a microcephaly case-control study conducted in Northeast Brazil (2016). Our results showed a strikingly high frequency of ZIKV exposure among mothers after the first wave of the ZIKV outbreak in this setting. Additionally, ZIKV and DENV immune status, as detected by the neutralization test, showed distinct patterns among pregnant women in this endemic area. We detected a low frequency of serological markers of recent ZIKV infection in samples collected just after delivery, highlighting the need for screening for ZIKV immune status in the early stage and throughout pregnancy to monitor congenital ZIKV syndrome.

## Introduction

Zika virus (ZIKV) is an arthropod-borne flavivirus closely related to several human pathogens of public health significance, including dengue viruses (DENV1-4), yellow fever virus (YFV), Japanese encephalitis virus (JEV) and West Nile virus (WNV) [1,2]. The emergence of ZIKV in the Pacific Islands and more recently in the Americas has been linked to an unprecedented range of neurological disorders and congenital syndrome, notably Guillain-Barré syndrome and fetal microcephaly [3–6]. Currently, autochthonous transmission of ZIKV has been reported in over 50 countries [7].

The countries most affected by extensive ZIKV outbreaks overlap with geographic areas where dengue has been endemic or hyperendemic [8,9]. Thus, a significant proportion of the population living in these regions has already been exposed to at least one DENV serotype [9]. ZIKV and DENV share a high degree of sequence identity and structural similarity [10], leading to potential immunological cross-reactivity [11,12].

To address the challenges in serology-based testing of flavivirus-immune patients, a considerable amount of effort has been made to establish reliable serological assays with the ability to discriminate ZIKV and DENV infections [13,14]. However, serological assays based on the detection of binding antibodies are difficult to interpret due to cross-reactivity, and virus neutralization tests need to be performed to rule out misleading positive serological results, especially in areas where past/current DENV circulation has been recorded [14–19].

The plaque reduction neutralization test (PRNT) measures a functional subset of virus-specific neutralizing antibodies (NAbs) and, consequently, is more specific than antibody binding assays [20,21]. PRNT remains the gold standard test to distinguish between previous flavivirus infection and to discriminate different DENV serotypes. However, virus neutralization assays are time-consuming, and appropriate laboratorial infrastructure and skilled technical staff are required to perform the tests [14,20,21]. Moreover, transient cross-neutralization might also contribute to difficult result interpretation when testing samples collected during or soon after recovery from individuals experiencing a secondary flavivirus infection [15,16,18,19].

Brazil experienced the largest ZIKV epidemic in the Americas and was the first country to report an increase in the prevalence of microcephaly associated with ZIKV infection [22]. The northeast region, the second most populous area of Brazil, accounted for approximately one third of the notified ZIKV cases [23] and for the vast majority of the microcephaly cases reported in the country [24]. The reasons for the higher incidence of ZIKV infections and microcephaly in this region remain unclear. Additionally, the population exposure to ZIKV after the first outbreak in the northeast has not yet been fully explored.

We have previously reported a case-control study showing a causal link between microcephaly at birth and congenital ZIKV infection [25]. Moreover, we demonstrated a high frequency of ZIKV-specific NAbs on the mothers of cases and controls [25]. Here, well-characterized samples of this case-control study were used to explore in-depth ZIKV and DENV NAbs profile among the mothers of cases and controls according to their previous dengue exposure. In addition, we described the dynamics of the transplacental transfer of ZIKV and DENV antibodies.

## Methods

### Study population and design

This study was part of a case-control study carried out in the city of Recife (Pernambuco state, Northeast Brazil), between January and December 2016 [25], after the peak of the microcephaly epidemic (November 2015) in this region [24]. DENV was first detected in Pernambuco State in the early 1980s [26,27], while autochthonous transmission of Zika and chikungunya viruses was reported in early 2015 [28]. Yellow fever and dengue vaccines are not recommended or publicly available in this setting. Since 2017, transmission risk of YFV has been a concern in some Brazilian states due to clustered outbreaks, mainly in the South and Southeast regions, where YFV vaccination is currently recommended for residents and travelers. Pernambuco state has not been included in the current YFV risk areas by the Pan American Health Organization (PAHO) [29].

Details of the study design, inclusion/exclusion criteria and data collection of the case-control study have been previously described elsewhere [25]. Cases were defined as newborns (alive or deceased) with head circumference of 2 standard deviations (SD) below the mean for sex and gestational age in the appropriate chart. Controls were live newborns without microcephaly and with normal brain imaging by transfontanellar ultrasonography (USG). For each microcephaly case, two controls were selected and matched by area of residence and expected date of delivery. Mothers and their newborns were consecutively recruited at birth in eight public maternity units in the metropolitan region of Recife. Mothers were interviewed by trained nurses using a structured standardized questionnaire.

### Study samples and definition of ZIKV and DENV infection

We included 89 out of 91 mothers of cases (two mothers without biological specimens) and 173 controls. Maternal blood samples were drawn solely at the time of admission for delivery, and no mothers reported febrile illness. For all neonates, umbilical cord blood was collected, and cerebrospinal fluid (CSF) was collected only for cases. These samples were collected immediately after birth. Serum samples were separated and stored at -70°C until tested.

For the mothers, we considered recent ZIKV infection if the subject tested positive with a ZIKV RNA test using real-time quantitative reverse transcription polymerase chain reaction (qRT-PCR) and/or for ZIKV-specific immunoglobulin M (IgM) and/or immunoglobulin G subclass 3 (IgG3) anti-NS1 (nonstructural protein 1) antibodies by enzyme-linked immunosorbent assay (ELISA). Positivity for ZIKV-specific IgG3 anti-NS1 indicates extended recent infection herein interpreted as recency of infection (IgG3 anti-NS1 antibodies are detectable for 4 to 6 months after infection [30]). Previous exposure to ZIKV and/or DENV exposure was confirmed if mothers tested positive for ZIKV and/or DENV1-4 NAbs by PRNT. In addition, the following classifications were used: (i) naïve population, NAbs for ZIKV or DENV were undetectable (antibody titers <1:20); (ii) single flavivirus infection, mothers tested positive for only one flavivirus infection (antibody titers >1:20 for only one virus); (iii) multiple flavivirus infections, characterized by the detection of NAbs (titers >1:20) for more than one virus.

We defined laboratory-confirmed microcephaly neonates as those who tested positive for the detection of the ZIKV genome by qRT-PCR and/or ZIKV-specific IgM by ELISA in any biological specimen (umbilical cord blood and/or CSF) at birth.

### Ethical issues

Written informed consent was obtained from a parent or guardian of each parturient or healthy infant enrolled in the study. The protocol for the study was approved by the Research Ethics Committee of the Pan American Health Organization (PAHO-2015-12-0075) and Aggeu Magalhães Institute, Oswaldo Cruz Foundation (IAM/ FIOCRUZ-PE) (CAAE: 51849215.9.0000.5190).

### Laboratory procedures

Serum samples of mothers and neonates (cases and controls) and CSF of neonates (cases) were tested by qRT-PCR for the detection of the ZIKV genome and IgM capture ELISA for the detection of ZIKV-specific IgM antibodies. For qRT-PCR, virus RNA was extracted using the QIAamp Viral RNA Mini Kit (Qiagen, Valencia, CA) following the manufacturer’s instructions. One-step qRT-PCR was performed using primers and probes described by Lanciotti and colleagues [15].

IgM-capture ELISA was conducted using a protocol previously described [31] and reagents were provided by the US Centers for Disease Control and Prevention (CDC; Fort Collins, CO, USA). To account for potential flavivirus cross-reactivity, samples were tested in parallel with ZIKV and DENV antigens. ZIKV antigen (CDC Vero E6–derived, inactivated ZIKV antigen (whole virus); virus strain H/PF/2013) and normal antigen (CDC Vero E6–derived, mock-infected normal antigen) were kindly provided by CDC. DENV antigen, a mixture of the 4 DENV serotypes, was prepared in a similar fashion using virus strains isolated in the study setting (Recife, Northeast Brazil): DENV-1 (BR-PE/97-42735), DENV-2 (BR-PE/95-3808), DENV-3 (BR-PE/02-95016), and DENV-4 (BR-PE/12-008). Positive (CDC humanized 6B6C-1 pan-flavivirus) and negative (pooled flavivirus-negative serum) controls were included in each plate. All sera were tested in duplicate, and the results were calculated as a ratio of the average optical density (OD) value of the test sample (P) divided by the average OD value of the negative control (N). P/N values of <2.0 were considered negative; >3.0, positive; and 2.0–3.0, equivocal. Samples showing positive results for both Zika and dengue antigens were considered positive for ZIKV IgM only if the ZIKV P/N ratio was at least twice the DENV P/N ratio [31].

Maternal sera were additionally tested by a novel *in-house* ELISA for the detection of ZIKV-specific IgG3 anti-NS1 antibodies, following a protocol described in details elsewhere [30]. Serum samples, in duplicate, were tested in parallel using purified NS1 proteins from Zika and DENV1-4 as antigens. Recombinant NS1 proteins expressed in the mammalian cell line 293 (Native Antigen Company, Oxfordshire, UK) included ZIKV NS1 (strain Uganda/MR/766) and DENV-1 (strain Nauru/Western Pacific/1974), DENV-2 (strain Thailand/16681/84), DENV-3 (strain Sri Lanka D3/H/IMTSSASRI/2000/1266) and DENV-4 (strain Sri Lanka D3/H/IMTSSA-SRI/2000/1266) NS1. Assay controls included: Zika positive (sera from convalescent patients collected 60 days post onset of symptoms); dengue positive recent infection (pooled sera from early convalescent virologically and/or serologically confirmed dengue patients, collected 20–30 days post onset of symptoms); and flavivirus-naïve sera (human type AB serum from healthy individuals from USA [MP Biomedicals, Solon, USA] diluted in IgG depleted human serum [Molecular Innovation, Novi, USA]). For ZIKV IgG3 analysis, the results were calculated as a ratio by dividing the average OD value of the test sample by the average OD value of the dengue recent infection control. The cut-off value for ZIKV IgG3 anti-NS1 antibodies positivity was based on a ratio>1.2 [30]. Positive ZIKV IgG3 anti-NS1 results were interpreted as recent ZIKV infections.

ZIKV and DENV1-4 NAbs were assessed by PRNT in maternal and neonate sera following a standardized protocol [30–32]. Briefly, PRNT was conducted in Vero cells seeded at a density of 300,000 cells/mL using 24-well plates. Serum samples were heat-inactivated (30 minutes at 56°C), serially diluted (4-fold dilution, starting at 1:20) and mixed with ≈30–100 plaque-forming units (PFU) of each challenge virus. Virus strains used in the assay were isolated in the study setting (Recife, Northeast Brazil): ZIKV (BR-PE243/2015), DENV-1 (BR-PE/97-42735), DENV-2 (BR-PE/95-3808), DENV-3 (BR-PE/02-95016), and DENV-4 (BR-PE/12-008) [31,32]. The virus-serum mixtures were incubated for 1 h at 37°C and then transferred to a monolayer of Vero cells to allow virus adsorption. After incubation, cells were covered with semisolid medium and incubated for 6–7 days at 37°C. Next, the cell monolayer was fixed with formalin solution and stained with crystal violet. The plates were washed and allowed to dry before plaque counts. The cutoff value for PRNT positivity was defined based on a 50% reduction in plaque counts (PRNT_50_) in the lowest dilution tested (1:20). Final titer calculation does not take into account the dilution made by mixing virus and antibody dilution (1:1 volume ratio). To estimate virus-specific NAb titers (log 10 transformed), we calculated IC_50_ values by nonlinear regression using the sigmoidal dose response (variable slope) equation on GraphPad Prism 7.0a. Serum samples were considered positive for each virus tested when antibody titers were >1:20 dilution. The PRNT assay for ZIKV has been validated using a well-characterized panel of longitudinal serum samples from primary and secondary flavivirus-infected individuals collected before and after ZIKV introduction in Brazil [30]. All laboratory procedures were conducted at the Virology Department (LaViTe) of the Aggeu Magalhães Institute, Oswaldo Cruz Foundation (IAM/ FIOCRUZ-PE).

### Data analysis

Statistical analysis was performed using SPSS software (version 12) and Graph Pad Prism (version 7.0a). We described the frequency of recent ZIKV infection and ZIKV and dengue serotype-specific (DENV1-4) NAbs among mothers of cases and controls. Serological profiles among mothers of cases and controls were analyzed using a conditional logistic regression. The stratified analysis of the association between maternal ZIKV positivity (NAbs) and previous dengue exposure was performed using the Mantel-Haenszel test. We applied a dot plot to show the NAbs titer distribution of mothers of cases and controls according to their previous dengue immune status. Antibody titers were compared between groups using a nonparametric Mann-Whitney test. We further explored the distribution of ZIKV NAbs titers among mothers according to two categories of microcephaly cases (ZIKV laboratory-confirmed and laboratory-negative cases) and the control group. For all comparisons of maternal antibody titers, we included 89 cases and 89 controls. The frequency of ZIKV exposure by these categories was analyzed by a chi-square test for linear trend.

We performed principal components analysis (PCA) to identify patterns and simplify structures underlying the multiple markers of recent or previous ZIKV and DENV infection among mothers, to identify groups of variables that were mainly correlated with each component, and to calculate individual scores related to each one of these components. The explanatory variables used were ZIKV-specific IgM and IgG3 anti-NS1 antibodies and ZIKV, DENV-3 and DENV-4 NAbs. The Kaiser-Meyer-Olkin (KMO) test was used to assess the measure of sampling adequacy. Bartlett’s test of sphericity was also applied to verify the sufficiency of the correlation between the variables for the PCA analysis, where a nonsignificant result (*p*>0.05) would indicate a lack of suitability of the variables for identifying underlying components. In the first step of PCA, we retained factors in the model with an eigenvalue ≥ 1.0. The second step was to identify variables strongly correlated with each component. These coefficients were the factor loadings generated in the component matrix. The individual factor scores for each component were transformed to a 0 to 1 scale and then added to the dataset. The dependent variable was the neonates’ status (microcephaly case or control), and the difference between the factor scores within these groups was assessed by the Wilcoxon nonparametric test for two independent samples. For the principal component analysis, we did not take into account correlation.

For the analysis of placental transfer, maternal and newborn antibodies mean titers were compared using the nonparametric Wilcoxon test for paired samples. The placental transfer ratio (TR) was calculated as follows: TR = [newborn antibody titer/maternal antibody titer] × 100. The median TRs of ZIKV antibodies between cases and controls were compared using the Mann-Whitney test. For the comparisons of TRs between ZIKV, DENV-3 and DENV-4 groups, we used a nonparametric Kruskal-Wallis test. Pearson correlation was used to measure the association between maternal levels and transfer ratio (TR) of ZIKV antibodies to the newborn. The level of significance was set at 0.05.

## Results

A total of 262 mothers of 89 neonates born with microcephaly (cases) and 173 controls without microcephaly were analyzed. The mean maternal age was similar between the cases and control groups. At the time of delivery, no mothers had positive ZIKV RNA tests (qRT-PCR). Overall, 7.25% of the mothers were positive for recent ZIKV infection by IgM and/or IgG3 anti-NS1 antibodies. Only 5.6% of the mothers of cases and 1.7% of the control group were positive for ZIKV-specific IgM. Considering recency of infection, 8.0% and 3.5% were positive for IgG3 anti-NS1 for mothers of cases and controls, respectively, but these differences were not statically significant. Overall, 61.4% of the participants had ZIKV infection (based on NAbs) independent of their dengue immune status. ZIKV infection detection by PRNT was slightly higher among mothers of cases (69.6%; 95%CI 59.4–78.1) than that of mothers of controls (57.2%; 95%CI 49.7–64.3) (p = 0.054) (Table 1).

**Table 1:** Characteristics of the mothers enrolled on the case-control study.

Characteristics	Mothers of cases (n = 89)	Mothers of controls (n = 173)	p value^a^
Age, mean ±SD	26.6±8.4	25.1±6.8	0.066
Recent ZIKV infection			
*ZIKV RNA test (qRT-PCR)*	0 (0.0%)	0 (0.0%)	-
*IgM antibodies (capture-ELISA)*	5 (5.6%)	3 (1.7%)	0.098
*IgG3 anti-NS1 (ELISA)*^*b*^	7 (8.0%)	6 (3.5%)	0.139
ZIKV infection status			
*Neutralization test*	62 (69.6%)	99 (57.2%)	0.054
*Flavivirus*-specific NAbs profile			
*Multiple Flavivirus infection*	72 (80.9)	143 (82.7)	0.931^c^
ZIKV/ DENV-3/ DENV-4	48	81	
DENV-3/ DENV-4	16	52	
Others virus combinations	8	10	
*Single Flavivirus infection*	9 (10.1)	16 (9.2)	0.727^c^
ZIKV	7	8	
DENV-3	-	4	
DENV-4	2	4	
*Flavivirus (ZIKV/ DENV) naïve*	8 (9.0)	14 (8.1)	

Abbreviations: ZIKV, Zika virus; RNA, Ribonucleic acid; qRT-PCR, real-time quantitative reverse transcription polymerase chain reaction; IgM, immunoglobulin M; ELISA, enzyme-linked immunosorbent assay; IgG3, immunoglobulin G subclass 3; NS1, nonstructural protein 1; NAbs, neutralizing antibodies; DENV, dengue virus

^a^ Conditional logistic regression

^b^ Not done for 1 case and 4 controls

^c^ Compared to Flavivirus (ZIKV/DENV) naïve

Table 1 also shows the profile of flavivirus-specific NAbs for the mothers. The majority of the mothers of cases (80.9%) and controls (82.7%) had NAbs to multiple flaviviruses, predominately for the combination of ZIKV, DENV-3 and DENV-4. The overall frequency of mothers with NAbs to DENV-1 and DENV-2 was lower than 3%. ZIKV was the single flavivirus infection in 7.8% (7/89) of the mothers of cases and 4.6% (8/173) of controls. Eight mothers of cases (8/89) and 14 (14/173) mothers of controls did not have detectable antibodies for ZIKV or DENV exposure.

**Fig 1:**
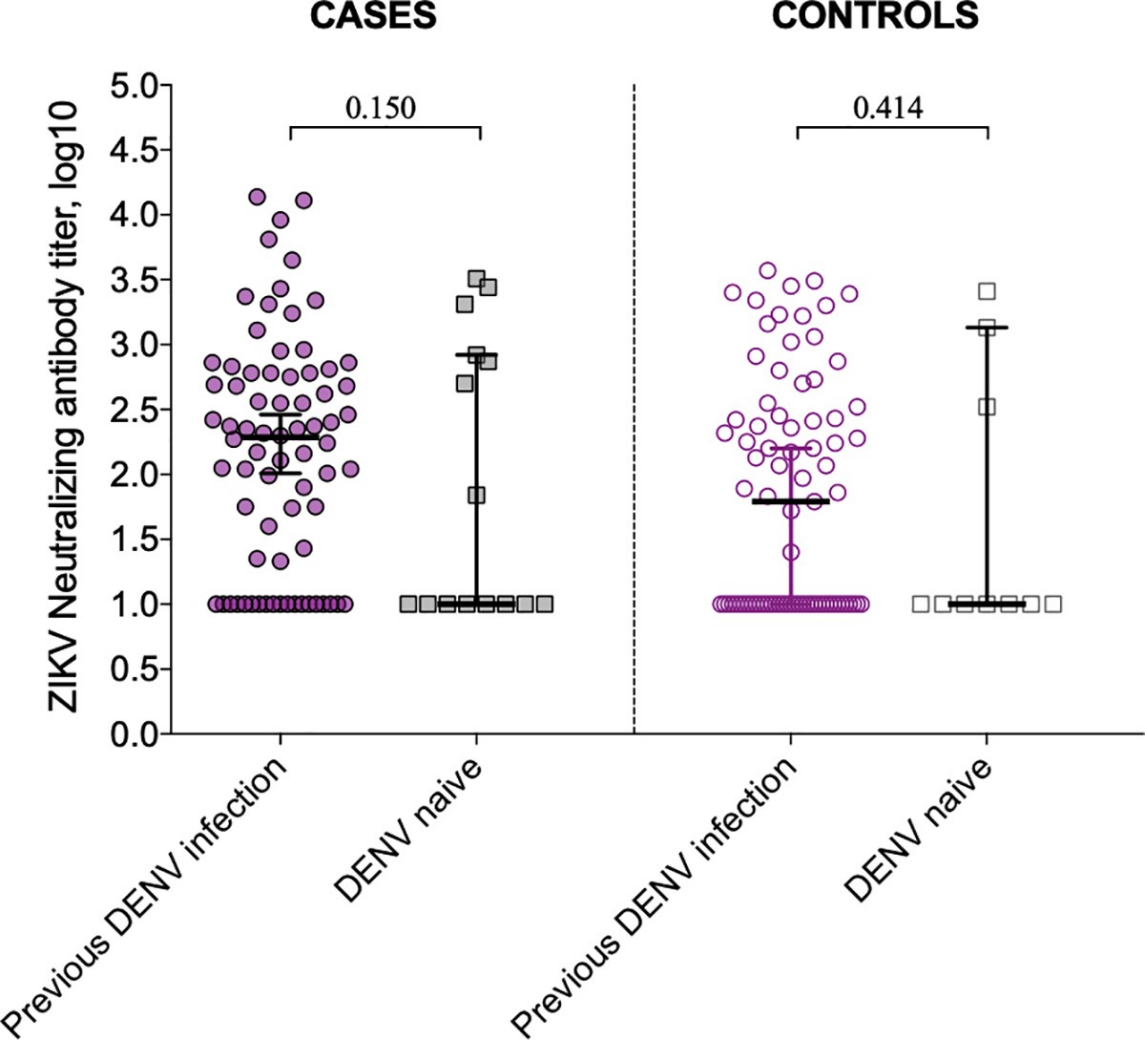
ZIKV-specific NAbs titers (log10) among mothers of cases and controls according to previous DENV exposure.

Overall, mothers with serological markers of exposure to DENV infection were more likely to have detectable levels of NAbs to ZIKV than dengue-naïve mothers (64.9% vs. 40.6%, respectively; RR: 1.60; 95%CI 1.07–2.39; p = 0.006) (Table 2). This higher frequency of ZIKV positivity among mothers exposed to dengue was also observed when analyzing by mothers of cases (74.3% vs. 46.7%; p = 0.033) and controls (60.2% vs. 36.3%; p = 0.034). Fig 1 shows the ZIKV-specific NAbs titers among mothers of cases and controls according to their DENV exposure status. The mean titers of ZIKV NAbs antibodies between DENV-naïve and DENV-infected mothers were similar for mothers of cases (p = 0.150) or controls (p = 0.414).

**Table 2:** ZIKV positivity by PRNT according to DENV exposure in mothers of cases and controls.

Previous DENV exposure	ZIKV NAbs		Total
	Positive	Negative	
*DENV immune*	146 (64.89%)	79 (35.11%)	225
*Naïve*	15 (40.54%)	22 (59.46%)	37
Total	161	101	262

Abbreviations: DENV, dengue virus; ZIKV, Zika virus; NAbs, neutralizing antibodies

*χ*^2^ Mantel-Haenszel = 7.7; Overall OR = 2.9; p value = 0.006

**Fig 2:**
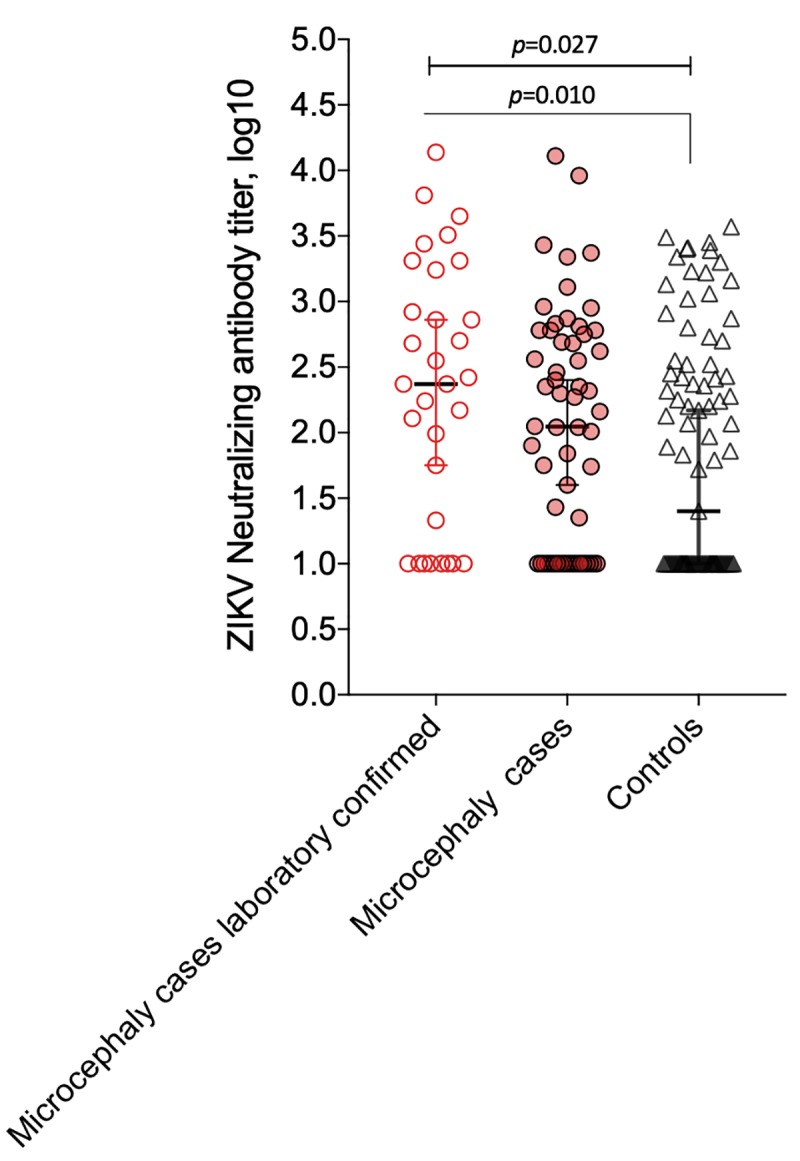
ZIKV-specific NAbs titers (log10) among mothers of ZIKV laboratory-confirmed microcephaly cases, microcephaly cases and controls. Microcephaly cases ZIKV laboratory-confirmed by qRT-PCR and/or ZIKV-specific IgM (n = 31), microcephaly cases with negative results by qRT-PCR and/or ZIKV-specific IgM (n = 58) and controls without microcephaly (n = 89).

Higher levels of ZIKV NAbs titers were detected among mothers of microcephaly neonates with laboratory confirmation of recent ZIKV infection (qRT-PCR and/or ZIKV-specific IgM) (n = 31) when compared to that of mothers of controls without microcephaly (*p* = 0.010; Fig 2). Additionally, there was a statistically significant trend of positivity for ZIKV NAbs when considering mothers of laboratory-confirmed microcephaly cases (OR = 2.84), mothers of microcephaly cases not laboratory-confirmed (OR = 1.22) and controls (chi-square for trend: 4.90; p = 0.026).

**Fig 3:**
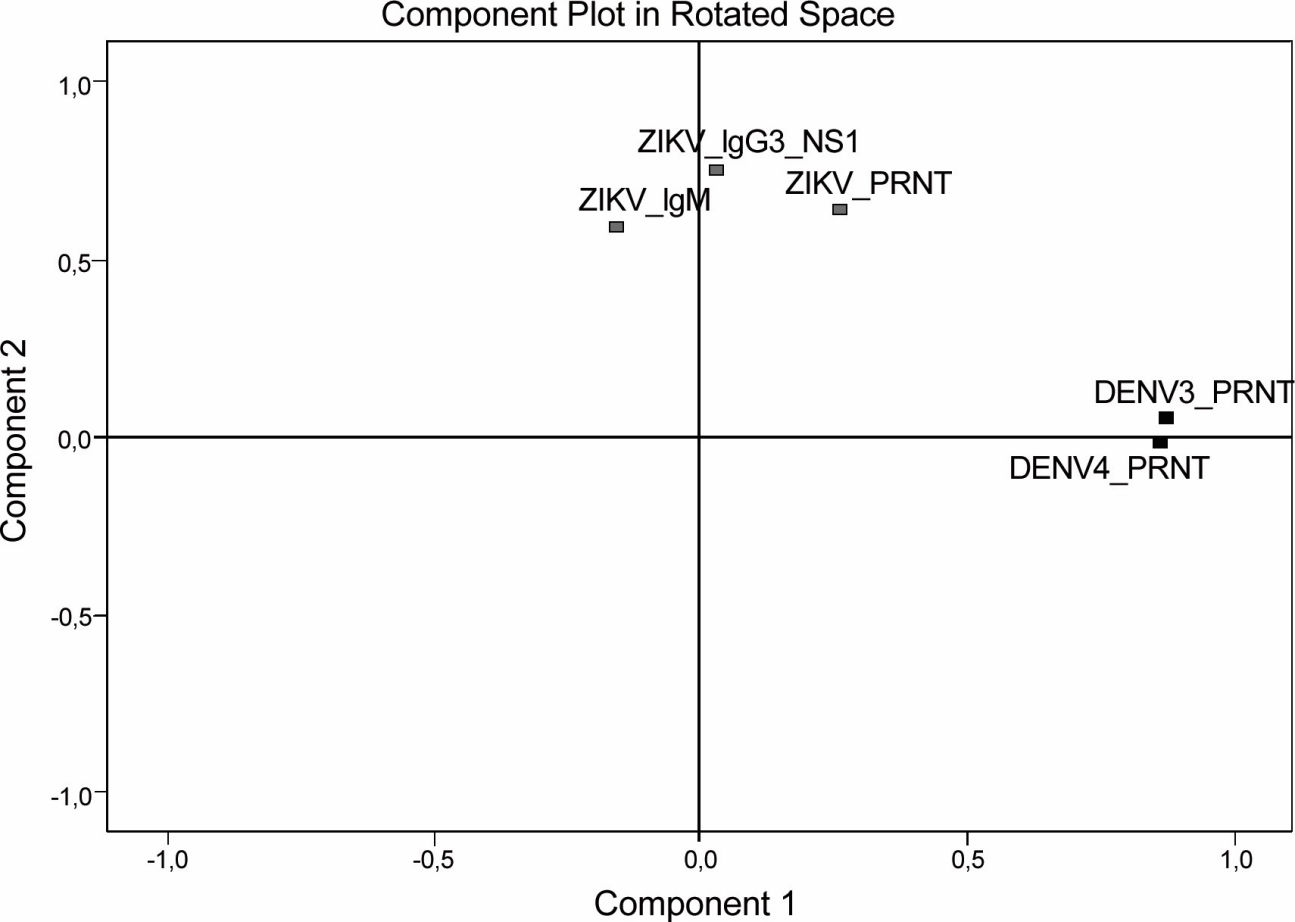
Principal component analysis (PCA) using ZIKV and DENV serological assays as explanatory variables.

Considering the PCA, the KMO test result was 0.530, and Bartlett’s test was significant (p< 0.0001), which indicated a sufficient correlation between the variables to perform the analysis. A two-factor model emerged from the data reduction when the criteria of eigenvalues >1.0 were applied. This two-factor model accounted for 59% of the total variation. After varimax rotation, component 1 presented the variables strongly correlated with previous DENV infection, and component 2 presented the variables strongly correlated with recent and previous ZIKV infection (Fig 3). For each individual, we calculated the scores related to each component. For component 1, there was no significant difference between means of mothers of cases (mean rank: 127.1) compared to those of mothers of controls (mean rank: 129.9; p = 0.768). For component 2, the scores of recent and past ZIKV infection were higher among mothers of cases (mean rank: 142.7) than those of mothers of the controls (mean rank: 121.9; p = 0.033).

**Fig 4:**
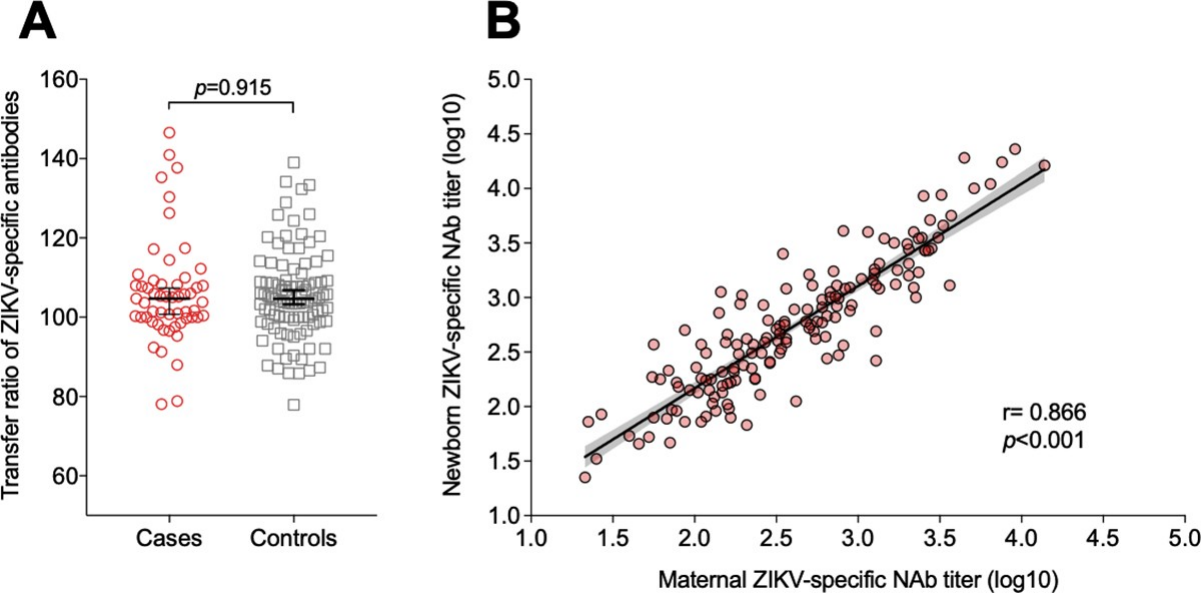
Placental transfer ratio (TR) of ZIKV-specific antibodies. A, Transfer ratio of Zika specific NAbs according to cases and controls; B, Correlation between ZIKV NAbs titers in the maternal and newborn samples.

Table 3 and Fig 4 show the placental transfer of ZIKV-, DENV-3- and DENV-4-specific antibodies in the mother-neonate pairs. ZIKV-specific NAbs levels were significantly higher in the neonates than in the corresponding mothers (TR: 104.7%; p<0.001). A similar pattern was observed for the levels of DENV-3- (TR: 109.1%; p<0.001) and DENV-4- (TR: 107.9%; p<0.001) specific antibodies. Dengue serotype-specific NAbs were more efficiently transferred through the placenta than ZIKV antibodies (DENV-3>DENV-4>ZIKV; p<0.001). The placental transfer ratio of ZIKV antibodies to the neonates did not differ between cases and controls (p = 0.915; Fig 4A). We found a positive correlation between ZIKV antibody titers in the maternal and newborn samples (r = 0.866; p<0.001). This serological pattern was similar between cases (r = 0.8994; p<0.001) and controls (r = 0.8432; p<0.001; Fig 4B).

**Table 3:** Placental transfer ratio (TR) of ZIKV and DENV specific antibodies in 254 (Cases = 81; Controls = 173) mother-neonates paired samples.

Serologic variable	Positive mothers, n (%)	NAbs titers^a^, median (range)		TR^b^	p value^c^
		Mother	Newborn		
ZIKV	156 (61.4)	2.55 (1.33–4.14)	2.69 (1.35–4.36)	104.7	<0.001
DENV-3	205 (80.7)	2.52 (1.35–3.66)	2.79 (1.43–4.06)	109.1	<0.001
DENV-4	205 (80.7)	2.48 (1.47–3.57)	2.64 (1.64–3.67)	107.9	<0.001

Abbreviations: NAbs, neutralizing antibodies; TR, transfer ratio; ZIKV, Zika virus; DENV, dengue virus

^a^ Antibody titer were log transformed (log10)

^b^ Transfer ratio = (newborn antibody titer/maternal antibody titer) x 100

^c^ Wilcoxon test (paired samples)

## Discussion

The emergence of ZIKV in the Americas and its cocirculation in dengue endemic areas has hampered the diagnosis of flavivirus infections [14]. The potential for cross-reactivity of antibodies induced by these viruses has complicated serological confirmation of Zika and dengue infections, requiring cumbersome confirmatory assays such as neutralization tests [11,12,15–18,20]. In this study, we used a large panel of serum samples from a case-control study to unveil the ZIKV and DENV immune profiles of mothers after the first wave of ZIKV transmission in a previously unexposed population. The findings reported inherently reflect the antibody profile at the time of delivery of mothers of microcephaly cases and controls living in the epicenter of the microcephaly epidemic, which is also a high DENV transmission area [26,27].

At the time of delivery, mothers had a low frequency (~8%) of positivity for ZIKV-specific IgM and a novel IgG3 anti-NS1 assay, the latter measuring recency of Zika exposure in the past six months [30]. In a previous publication, our group reported 31% recent ZIKV infection (IgM) among mothers of microcephalic infants in the same setting [31,33]. One of the possible explanations for the variations in the incidence of ZIKV infection among mothers is the different microcephaly inclusion criteria between studies. In the initial publication, when the etiological cause of congenital microcephaly was under investigation, the case series included only severely microcephalic infants recruited at the early stage of the outbreak [31,33]. Nevertheless, both studies showed that at delivery markers of recent ZIKV infection among mothers might not be detectable. These findings support the waning of IgM antibodies over time [34], suggesting a lag time between virus exposure and time of delivery. In fact, higher levels of ZIKV NAbs titers among mothers of microcephalic neonates with laboratory confirmation of recent ZIKV infection are consistent with a more recent infection. However, neither the case-series nor the case-control study allows for the assessment of the timing of ZIKV infection among mothers of microcephaly cases. These issues related to the timing of ZIKV infection and development of adverse outcomes during pregnancy can be further investigated in ongoing cohort studies.

Notably, our findings demonstrated a strikingly high prevalence of ZIKV exposure, as defined by PRNT, in the study population close to the peak of the microcephaly outbreak in Northeast Brazil. We found that approximately 60% of the mothers had already been infected by ZIKV, suggesting a high transmission rate in a short period of time (2015–2016) after the introduction of ZIKV into this naïve population. This finding further supports the information of high exposure rates from previous Zika outbreaks in Micronesia [35], French Polynesia [36], Brazil [30,37] and other Latin America countries [38]. Notably, the low proportion of ZIKV-naïve women might explain the reduction in the number of cases of congenital Zika syndrome following the first large Zika outbreak in this setting [24].

Our results also demonstrated a high prevalence of multiple flavivirus exposures among mothers of microcephaly cases and controls at birth. Our data confirmed that DENV-3 and DENV-4 serotypes were the predominant pre-exposure dengue NAbs, as reported by other studies conducted before [32] and after [25,33,39] the ZIKV outbreak in the same setting. The predominance of DENV-3 and DENV-4 serotypes in our study population reflects the epidemiological scenario of DENV circulation in the last decade in the study setting [26,27]. DENV-3 was the sole serotype circulating in Recife between 2002 and 2006. DENV-4 predominantly circulated in the setting after its introduction in 2010. DENV-1 and DENV-2 circulated in the city in the 1990s before DENV-3 introduction. Data from different epidemiological studies conducted by our group and from the surveillance system of the city registered lower rates (nonepidemic) of DENV-1 and DENV-2 circulation between 2012–2014; however, these viruses were quickly displaced by the introduction and circulation of ZIKV and CHIKV in the setting [40]. Although immunity to DENV-1 and DENV-2 was present among the DENV-immune mothers, it represented only ~3% of the participants, and we concentrated our analysis on DENV-3 and DENV-4 serotypes. We acknowledge that this lower frequency of DENV-1 and DENV-2 serotypes might be related to the sensitivity of the assay to detect lower levels of NAbs (<1:20), considering that these viruses circulated predominantly in the 1990s in the study setting. However, our study population was comprised of mostly young mothers (~25 years old) who had not been exposed to DENV-1 and DENV-2 outbreaks in the setting. We also acknowledge that cross-reactions might complicate PRNT results interpretation and that multiple viruses positivity might, at some extent, represent some levels of cross-reactivity. However, the pattern of DENV serotype-specific profile found in our study matches the epidemiological scenario of DENV circulation in the setting. Our finding of high frequency of previous dengue exposure among mothers is in consonance with a recently published retrospective case-control study in the neighboring state of Paraiba [41]. Notably, we found that ~10% of the mothers of microcephaly cases did not show any serological markers of exposure to DENV and ZIKV by PRNT, which may be related to the sensitivity of the assay at the serum dilution used (1:20). We acknowledge that this frequency of ZIKV negatives might also represent mothers of microcephaly cases not associated with ZIKV infection during pregnancy. Prior to the Zika epidemic (2000–2014), the annual prevalence of microcephaly cases was 5.0 (CI: 3.6–6.6) per 100,000 live births, which corresponds to an annual average number of 44 cases in the Northeast region [42]. Considering this previous prevalence and the population sampled (13,531 live births), the expected number of non-ZIKV microcephaly cases would be 0.7 cases [25]. In 2015, the ZIKV epidemic year in the Northeast region, there were 1,142 registered microcephaly cases and a prevalence of 138.7 (CI: 130.9–147.0) per 100,000 live births [42], which represents a striking increase of the prevalence.

Notably, we observed a high frequency of ZIKV positivity among DENV-immune mothers. This finding might reflect the high risk of exposure of this population to *Aedes aegypti* and, consequently, to arthropod-borne virus infection in general [26,27,40]. Additionally, the antibody-dependent enhancement (ADE) phenomenon may play a role in increasing the risk of ZIKV severe outcomes among individuals previously exposed to DENV [43–45]. The relationship between dengue and Zika cross-reactive antibody interactions may be complex: high dengue antibody titers might provide some protection against Zika infection, while low titers might favor enhancement. However, we acknowledge that case-control studies are not the proper epidemiological study design to accurately address this question. Ongoing prospective cohort studies may provide more conclusive responses in the near future.

The main concern regarding serological assays is the potential for cross-reactivity of antibodies induced by DENV and ZIKV, posing a challenge in accurately differentiating infection by these viruses [11–18,20]. In our study, PCA using ZIKV and DENV serological assays as explanatory variables identified two main components: one strongly correlated with previous DENV infection and the other correlated with recent and previous ZIKV infection. Although most mothers of cases and controls included in our study had previous exposure to multiple flavivirus infections, the PCA results showed that the two factors are relatively uncorrelated, therefore representing different components of information. In addition, the scores for the component related to recent and past ZIKV infection were higher among mothers of cases than controls. Recently, there has been a growing body of work focusing on characterizing Zika and dengue immunological cross-reactivity on both binding and neutralization assays. Cross-neutralizing antibodies after a secondary flavivirus infection, whether dengue or Zika, have been observed by our group [40] and others [18,19]. Recent studies have demonstrated transient induction of flavivirus cross-neutralizing antibodies soon after recovery from a ZIKV or DENV infection in individuals who experienced secondary flavivirus infections [18,19]. However, little to no cross-neutralization has been detected in late convalescent samples (>2 months) [18], confirming that ZIKV-specific NAbs develop after a ZIKV infection, even in the presence of pre-existing dengue exposure [17,18]. In a case-control study design, it is not possible to determine the time of maternal infection. Our finding of a high frequency of ZIKV markers (as determined by PRNT) and a low frequency of recent ZIKV infection suggest that there was a lag time between viral exposure and time of delivery. Hence, maternal samples collected at delivery potentially represent late convalescent serum, which probably explains the more specific neutralizing response for ZIKV observed in our study.

The efficient maternal-fetal transfer of ZIKV and DENV NAbs observed by our group is consistent with previous published studies from the same and other settings [33,39]. This finding reflects the active transport of IgG across the placenta, a well-documented immune mechanism mediated by the neonatal Fc receptor (FcRn), which is present on syncytiotrophoblast cells in the placental tissues [46]. Maternally acquired dengue-specific antibodies have been shown to play a dual role during infancy by first conferring protection at birth and then increasing the risk for severe dengue infection, as antibodies wane to subneutralizing levels [47]. Studies investigating the role of maternally transferred ZIKV antibodies in mediating protection or severe ZIKV or DENV disease during the first year of life are strongly needed. Additionally, data on the placental transfer ratio of dengue and Zika antibodies might be useful to infer the occurrence of congenital ZIKV infection in infants during the first year of life. In fact, the relative difference between maternal and infant ZIKV titers and maternal and infant DENV titers at later time points after birth (~6 months) have been used as a marker of ZIKV infection of the fetus [41]. Infants born to dengue-immune mothers usually have a sharp decline in DENV antibody titers, which were acquired through maternal transfer, at early ages. However, if infected during pregnancy, ZIKV antibody titers in the newborns will remain high as the infant produces antibodies to the in-utero ZIKV infection [41].

Our study reported in-depth Zika and dengue serological profiles in a well-designed case-control study carried out in a hyperendemic area of dengue transmission. The overall strength of our approach is the well-characterized antibody responses for ZIKV and DENV1-4 as measured by PRNT, which is normally not feasible in studies analyzing large sample panels. In summary, we detected a strikingly high frequency of ZIKV exposure among mothers during the first wave of the Zika outbreak in Northeast Brazil. In addition, the majority of the mothers were immune to multiple flavivirus infections. This information provides insights regarding the immune status of the population in relation to a recent ZIKV introduction and more than three decades of DENV circulation. Additionally, PCA suggests relatively independency between the set of variables related to ZIKV and DENV infections, confirming minimal cross-reactivity antibody interactions in the PRNT assay. Considering the relatively low frequency of markers of recent ZIKV exposure at delivery, screening for ZIKV immune status should be performed in the early stage and throughout pregnancy to monitor congenital ZIKV syndrome in endemic areas. Innovative laboratorial diagnostic approaches for ZIKV and DENV infections are urgently needed for the guidance of clinical practice and public health purposes.

## Supporting information

S1 Checklist STROBE checklist (case-control studies). (DOC)
